# Postpartum Septic Pelvic Thrombophlebitis of the Ovarian Vein in the Context of Acute Pyelonephritis: Clinical Challenges in the Decision to Anticoagulate

**DOI:** 10.7759/cureus.107893

**Published:** 2026-04-28

**Authors:** Carina McClean, Siya Bhutani, Shelby Watford, Nirmal Onteddu

**Affiliations:** 1 Medicine, University of Florida, Gainesville, USA; 2 Internal Medicine, University of Florida College of Medicine – Jacksonville, Jacksonville, USA

**Keywords:** anticoagulation, ovarian vein thrombosis, postpartum complication, sepsis, septic pelvic thrombophlebitis

## Abstract

Septic pelvic thrombophlebitis (SPT) is a rare postpartum complication that can be life-threatening if not promptly diagnosed and treated. We present the case of a 34-year-old woman who presented to the emergency department (ED) seven weeks postpartum with abdominal pain, nausea, vomiting, and diarrhea. Imaging revealed SPT with right ovarian vein thrombosis and concurrent acute pyelonephritis. The patient was found to be in septic shock requiring intensive care, broad-spectrum antibiotics, vasopressor support (norepinephrine) following 4.5 L of intravenous fluid resuscitation, and intravenous heparin. Initial workup was significant for urinalysis concerning infection and imaging showing a subtle filling defect with enlargement of the right ovarian vein with surrounding inflammatory changes. Despite negative blood cultures, she remained febrile following antibiotic change, prompting escalation to meropenem and daptomycin. Anticoagulation was initially started with therapeutic intravenous heparin, discontinued when the thrombus was considered potentially incidental, and later resumed as therapeutic enoxaparin at discharge for three months due to persistent thrombus burden and ongoing postpartum risk factors. This case highlights the diagnostic and therapeutic challenges of SPT, particularly when it presents alongside other infections such as pyelonephritis. In the absence of definitive guidelines, management decisions, including the use and duration of anticoagulation, must be individualized based on clinical status and thrombotic burden. Our case underscores the diagnostic overlap between pyelonephritis and SPT and the clinical challenges in determining when to initiate anticoagulation.

## Introduction

Septic pelvic thrombophlebitis (SPT) is a rare and potentially life-threatening disorder commonly associated with the postpartum period, which is defined as the first six to eight weeks after delivery. One study suggests the incidence is 1 in 3,000 deliveries [1]. SPT includes two main types - ovarian vein thrombophlebitis (OVT) and deep pelvic vein septic pelvic thrombophlebitis (DSPT), both of which can present with nonspecific symptoms, such as fever, acute-onset lower abdominal pain, and vaginal bleeding [2]. This presentation overlaps with that of conditions, such as appendicitis, pyelonephritis, or a pelvic abscess; therefore, diagnosis can be challenging. The postpartum period predisposes patients to thrombosis due to venous dilation, stasis from uterine enlargement, and a hypercoagulable state. Additional risk factors include maternal age less than 20 years, cesarean delivery, non-Hispanic Black race, multiple gestations, postpartum infections including endometritis (infection of the uterine lining) and chorioamnionitis (infection of amniotic fluid), underlying thrombophilia, postpartum blood transfusion, wound complications, hysterectomy, and repeat laparotomy [3]. Racial disparities in maternal outcomes likely reflect broader systemic inequities in access to care, social determinants of health, and structural factors, rather than inherent biological differences.

Imaging has a crucial role in the diagnosis of SPT. The first line for imaging is Doppler ultrasound of the pelvic and ovarian veins, whereas computed tomography (CT) or MRI help with diagnosis due to definitive visualization of these structures [4]. The first-line therapy of SPT includes early initiation of intravenous antibiotics to target pelvic pathogens.

However, the role of anticoagulation in SPT remains controversial. Although anticoagulation has been used to prevent thrombus formation and accelerate clinical recovery, there is no clear consensus regarding indications, choice of agent, or duration of therapy [5]. Some studies support anticoagulation to prevent thrombus propagation and expedite recovery, whereas others suggest antibiotics alone may be sufficient. There remains limited guidance on management when it occurs concurrently with other infections. We report a case of postpartum ovarian vein thrombosis in the setting of acute pyelonephritis, detailing the diagnostic overlap and evolving decision-making process surrounding anticoagulation in the absence of definitive management guidelines.

## Case presentation

Our patient is a 34-year-old gravida 9 para 6 woman who presented to the emergency department (ED) seven weeks postpartum for one-week progressive abdominal pain with associated nausea, vomiting, and diarrhea. She had a spontaneous vaginal delivery at 38 weeks of gestation, complicated by group B *Streptococcus *(GBS) colonization for which she received intrapartum clindamycin prophylaxis due to penicillin allergy. Six days before presentation, she received medroxyprogesterone. Her past medical history was significant for a previous molar pregnancy (G2), one preterm low transverse cesarean delivery due to non-reassuring fetal heart rate tracing (G6), one vaginal birth after cesarean section (G7), and anemia.

On presentation, the patient was in septic shock, with hypotension to 98/66 and tachycardia to 130 bpm, requiring aggressive fluid resuscitation (4.5 L) and vasopressor support with norepinephrine. Initial laboratory evaluation demonstrated marked leukocytosis with marked bandemia, metabolic acidosis, mild acute kidney injury, and elevated serum lactate (Table 1). The findings were consistent with acute pyelonephritis with pyuria, hematuria, and positive nitrites.

**Table 1:** Laboratory findings at admission, MICU, and discharge. CO₂: carbon dioxide, MICU: medical intensive care unit, WBC: white blood cell, ALT: alanine aminotransferase, AST: aspartate aminotransferase

Parameter	Admission	MICU	Discharge	Reference range
WBC (×10³/µL)	19.35	25.31	12.44	4.5-11
Bands (%)	38.1	21.2	6.1	0-10
Neutrophils Abs (×10³/µL)	15.53	22.4	9.06	1.40-7.50
Hemoglobin (g/dL)	10.3	9.6	7.8	12.0-16.0
Platelets (×10³/µL)	268	254	918	140-440
Creatinine (mg/dL)	1.08	1.06	0.62	0.51-0.96
CO₂ (mmol/L)	18	17	19	21-29
Lactic acid (mmol/L)	-	3.1	-	0.7-2.0
AST (IU/L)	38	131	20	14-33
ALT (IU/L)	45	87	18	10-42
Total bilirubin (mg/dL)	1	1.5	0.6	0.2-1.0
Albumin (g/dL)	3.2	2.8	3.2	3.8-4.9

**Figure 1:**
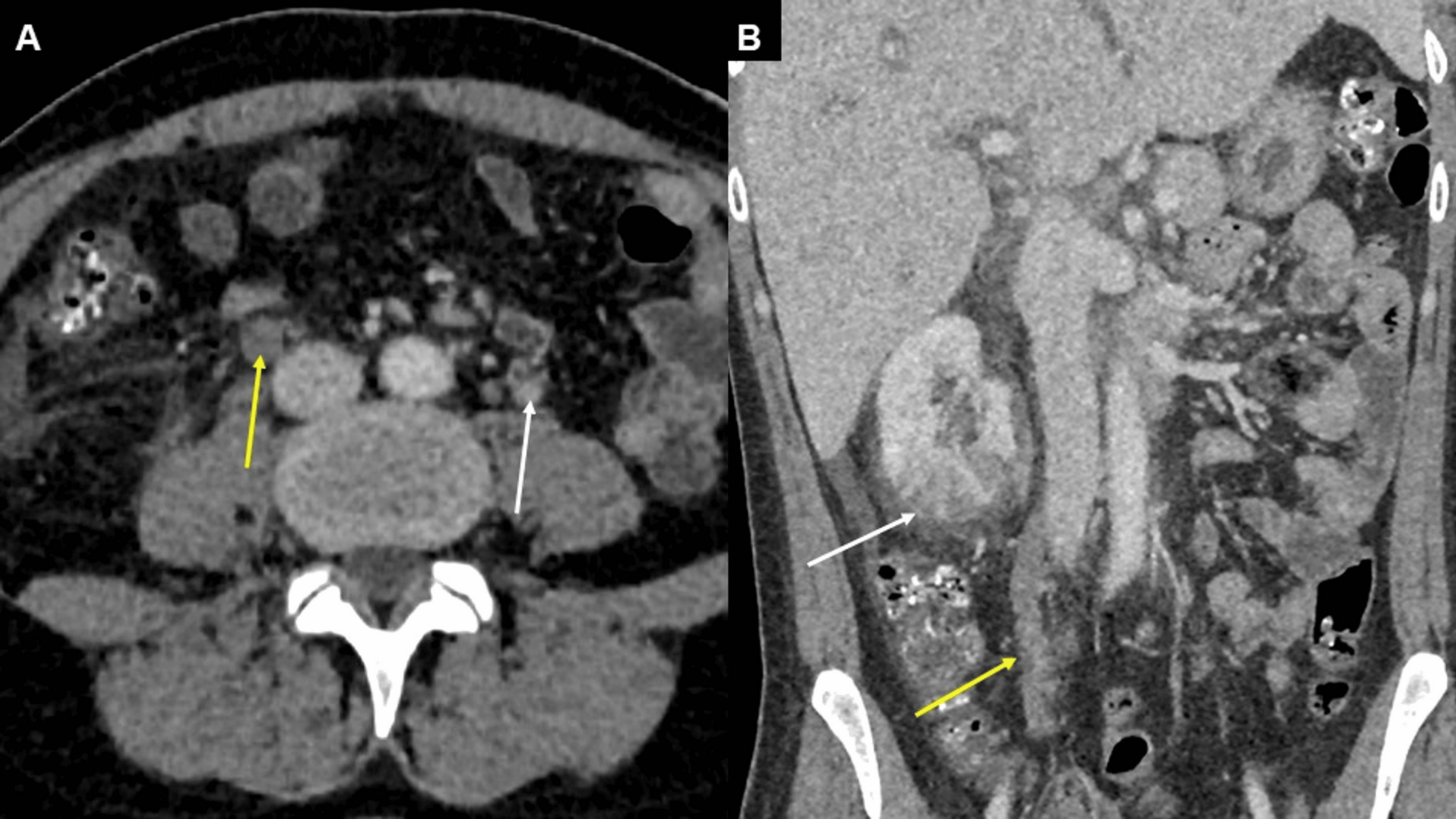
(A) Axial computed tomography of the abdomen with intravenous contrast in portal venous phase demonstrating asymmetrically prominent right ovarian vein with absent luminal contrast opacification (yellow arrow) and mild surrounding fat stranding. Note the normal opacification of the left ovarian vein (white arrow). (B) Coronal computed tomography again shows the absent contrast opacification of the right ovarian vein (yellow arrow), consistent with ovarian vein thrombosis in the appropriate clinical setting. Also note the striated right renal cortical enhancement with perinephric fat stranding (white arrow), representing acute pyelonephritis.

CT of the abdomen and pelvis revealed findings consistent with right-sided pyelonephritis, including perinephric fat stranding and enlargement of the right ovarian vein with an intraluminal filling defect, consistent with ovarian vein thrombosis. No renal vein thrombosis was identified. Additionally, imaging demonstrated marked intrahepatic biliary dilatation with moderate pericholecystic fluid, as shown in Figure 1. However, subsequent hepatobiliary iminodiacetic acid (HIDA) scan showed normal gallbladder function, and the patient’s transaminitis was attributed to ischemic hepatitis ("shock liver") in the setting of septic shock.

The patient was admitted to the medical intensive care unit (MICU) and was started on empiric broad-spectrum antimicrobial therapy with vancomycin, piperacillin/tazobactam, and therapeutic intravenous heparin infusion for suspected SPT with ovarian vein thrombosis. Blood and urine cultures remained negative throughout hospitalization. Following initial clinical improvement, therapeutic anticoagulation was discontinued after two days, as the ovarian vein thrombus was initially considered to be an incidental finding in the setting of severe infection. However, prophylactic enoxaparin was continued for venous thromboembolism prophylaxis.

Following the transition from piperacillin/tazobactam to oral amoxicillin/clavulanate, the patient developed a fever of 102°F and tachycardia. Repeat infectious workup remained negative. Given persistent fever and concern for resistant organisms, antimicrobials were broadened to piperacillin/tazobactam; however, fever persisted. Therefore, antibiotics were broadened further due to continued concerns for resistant organisms to vancomycin and meropenem, and subsequently to meropenem and daptomycin based on infectious disease consultation. Daptomycin was selected in the context of persistent clinical instability, need for broad gram-positive coverage, and considerations for outpatient parenteral antibiotic therapy. 

As the patient’s course evolved, repeat clinical assessment raised concern for ongoing thrombus burden in the setting of persistent postpartum hypercoagulability. After multidisciplinary reassessment involving hematology and infectious disease teams, therapeutic anticoagulation was re-initiated with enoxaparin before discharge.

The patient improved clinically and was transferred to the general medical floor. She was discharged back home after a nine day stay with a 14-day course of intravenous meropenem and daptomycin via a peripherally inserted central catheter (PICC) line, along with therapeutic enoxaparin for a planned duration of three months. The patient tolerated this treatment well with no further hospital admissions.

## Discussion

SPT is a rare but serious condition characterized by infected thrombosis of the pelvic veins [6]. Thrombophlebitis is an inflammatory process that causes a blood clot to form and obstruct one or more veins [7]. The affected veins may be near the surface of the skin or deep within the musculature, defined as superficial and deep thrombophlebitis, respectively [8]. The location of the clots varies and may involve the cerebral venous sinuses, internal jugular vein, pelvic veins, or be associated with catheter sites. The development of thrombophlebitis is closely linked to Virchow’s triad, which encompasses endothelial damage, venous stasis, and hypercoagulability [9]. In the postpartum period, ovarian vein dilation, uterine compression, and increased coagulability promote venous stasis and thrombosis, particularly in the right ovarian vein. In this case, the concurrent presentation of pyelonephritis and ovarian vein thrombosis posed a diagnostic and therapeutic challenge. The patient’s presentation with septic shock, abdominal pain, and fever could be fully explained by pyelonephritis alone, potentially delaying recognition of SPT. This emphasizes the importance of maintaining a high index of suspicion in postpartum patients with persistent severe infection.

Another notable feature in this case is the recent administration of depot medroxyprogesterone acetate. Although progestin-only contraceptives are generally considered to carry a lower risk of venous thromboembolism compared to estrogen-containing agents, their contribution in the setting of acute inflammation and sepsis remains uncertain. In this patient, the combination of postpartum physiology, active infection, and recent hormonal exposure may have contributed synergistically to thrombus formation.

The most significant clinical challenge in this case was the decision to initiate and maintain anticoagulation. Although antibiotics are the mainstay of treatment, there is no clear consensus regarding the addition of anticoagulants [10,11]. Historically, anticoagulation has been used to prevent thrombus propagation and embolization, and to expedite the resolution of symptoms, particularly in patients who do not show early clinical improvement on antibiotics alone [4]. Although some clinical studies have supported this approach, there are studies that also suggest many patients may recover fully with antibiotics alone, raising questions about the necessity and benefit of anticoagulation in all cases. The literature is heterogeneous and limited [12-14]. For instance, one study recommends that anticoagulation for ovarian vein thrombosis be the same length as for deep vein thrombosis, or around seven months. Others suggest a shorter course of anticoagulation, ranging from 7 to 10 days with IV heparin bridged to warfarin or up to three months in the presence of extensive thrombus. The anticoagulant of choice is similarly heterogeneous, with regimens involving low molecular weight heparin, unfractionated heparin, and transition to oral anticoagulants [4].

In our patient, anticoagulation decisions evolved over time highlighting this uncertainty. Therapeutic heparin was initially started due to concern for septic thrombophlebitis but was discontinued when the thrombus was considered potentially incidental in the setting of severe infection. However, with persistent clinical concern and recognition of ongoing postpartum hypercoagulability, anticoagulation was later resumed before discharge and to be completed for three months. This “start-stop-restart” approach reflects real-world clinical decision-making, where evolving clinical data and multidisciplinary input shape management. Enoxaparin was ultimately chosen due to its established safety profile in the postpartum period, predictable pharmacokinetics, and lower risk of heparin-induced thrombocytopenia compared to unfractionated heparin [12].

From an infectious standpoint, the common pathogens in postpartum pelvic infections and pyelonephritis include *Escherichia coli*, other Enterobacterales, streptococci, and anaerobes. Empiric therapy should target these organisms, with consideration of extended-spectrum beta-lactamase (ESBL) organisms in severe cases. Escalation to meropenem and daptomycin in this case reflects the challenge of persistent sepsis in the absence of microbiologic confirmation. Daptomycin is not standard therapy for pyelonephritis and highlights the balance between adequate antimicrobial coverage, stewardship consideration, and feasibility for outpatient parenteral therapy. According to the Infectious Diseases Society of America, empiric treatment for septic shock secondary to pyelonephritis should include broad-spectrum antibiotics targeting likely uropathogens, including streptococci, ESBL-producing enterobacterales, and anaerobes [9-11]. In this case, initial therapy consisted of piperacillin-tazobactam and vancomycin. This regimen was later adjusted to vancomycin and meropenem to provide enhanced coverage against ESBL-producing and other resistant organisms.

Overall, this case demonstrated the diagnostic complexity and therapeutic uncertainty associated with SPT, especially when it occurs concurrently with other infections. It highlights the need for heightened clinical suspicion, careful interpretation of imaging findings, and individualized management strategies, especially regarding anticoagulation.

## Conclusions

The decision to initiate anticoagulation should be individualized, taking into account the extent of thrombus, the severity of clinical symptoms, the presence of risk factors for thromboembolism, and the response to initial therapy. Clinicians should maintain a high index of suspicion for ovarian vein thrombosis in postpartum patients with persistent fever or septic shock, particularly when imaging findings overlap with pyelonephritis.

This case highlights the dynamic nature of anticoagulation decision-making, including initiation, interruption, and re-initiation. In the absence of large-scale randomized trials, physicians are forced to rely on clinical judgment and observational studies. Further studies are needed to better define treatment thresholds and guide evidence-based decision-making in the management of SPT.
